# Efficacy and Safety of Camrelizumab Monotherapy and Combination Therapy for Cancers: A Systematic Review and Meta-Analysis

**DOI:** 10.3389/fonc.2021.695512

**Published:** 2021-06-25

**Authors:** Jiting Wang, Song Su, Jun Li, Yaling Li

**Affiliations:** ^1^ Department of Pharmacy, Southwest Medical University, Luzhou, China; ^2^ Department of Pharmacy, Affiliated Hospital of Southwest Medical University, Luzhou, China; ^3^ Department of Hepatobiliary Surgery, Affiliated Hospital of Southwest Medical University, Luzhou, China; ^4^ Department of Anorectal, Affiliated Hospital of Southwest Medical University, Luzhou, China

**Keywords:** camrelizumab, efficacy, safety, monotherapy, combination therapy, meta-analysis

## Abstract

**Objective:**

This meta-analysis compared the safety and efficacy of camrelizumab monotherapy and combination therapy, aiming to provide a reference for the clinical combined use of camrelizumab in the treatment of cancers and also provide a reference for the development of subsequent indications of camrelizumab.

**Methods:**

Meta-analysis was used to analyze the four eligible literatures. Primary endpoints of effectiveness index were objective response rate (ORR), progression-free survival (PFS), control rate (CR). Primary endpoint of safety index was rating of severity of adverse drug reactions (grades 1–5).

**Results:**

The ORR, PFS, and CR values of combined treatment with camrelizumab was better than alone treatment, camrelizumab alone was better than chemotherapy (RR = 0.45; 95% CI, 0.30–0.67; P < 0.001; RR = 1.63; 95% CI, 1.25–2.13; P < 0.001; RR = 0.73; 95% CI, 0.52–1.02; P<0.001). When grade > 2, the incidence rate of combined treatment and chemotherapy are higher than monotherapy (RR = 0.66; 95% CI, 0.51–0.86; P<0.001). In any grade, the safety of camrelizumab combination therapy was better than that of monotherapy, the safety of chemotherapy was better than camrelizumab plus chemotherapy.

**Conclusion:**

In terms of effectiveness, the combination of camrelizumab is better than monotherapy, and monotherapy is better than chemotherapy. In terms of safety, when the grade > 2, single use is better than combination therapy and chemotherapy. In any grade of adverse event, the safety of combined use of camrelizumab is better than that of single use, and the safety of chemotherapy is better than the combined use of camrelizumab plus chemotherapy.

## Introduction

Malignant tumors have become a serious threat to human life and health, and it is on the rise in recent years. The treatment of tumors have developed from surgery, radiotherapy, chemotherapy, local treatment, and now to molecular targeted therapy, and quickly entered the era of immunotherapy, effectively extending the overall survival of patients (1–3). Tumor immunotherapy use immune blockade inhibitors, also known as immune checkpoint inhibitors (anti-programmed cell death 1 [PD-1] or anti-programmed cell death ligand 1 [PD-L1] inhibitor, ICI), are a new type of monoclonal antibody drug that can inhibit the function of inhibitory immune receptors in order to evoke an immune antineoplastic response (4). In recent years, with the success of pembrolizumab and nivolumab, which target PD-1, and atezolizumab, durvalumab, and avelumab, which target PD-L1, ICI have been extensively studied in various tumor types (5).

Camrelizumab (SHR-1210) is a humanized monoclonal antibody against PD-1 independently developed by China. It showed high affinity for PD-1 (KD = 3.31 nmol/L) and high receptor occupancy on circulating T lymphocytes (85% at a dose of 200 mg) (6). Phase 1 clinical trials showed that camrelizumab was well tolerated in patients with advanced solid tumors and showed antitumor activity (7–9). In China, camrelizumab has been approved for several indications including Hodgkin’s lymphoma (NHL), advanced hepatocellular carcinoma (HCC), non-small cell lung cancer (NSCLC), and esophageal cancer (EC) (10). In addition, many clinical trials have confirmed that camrelizumab has a significant effect on many other cancers, including nasopharyngeal carcinoma (NPC), gastric cancer, pancreatic cancer, renal cancer, cervical cancer, and so on (11, 12). Although it is known that camrelizumab has a clinical therapeutic effect on a variety of tumors and also has a good development prospect, whether monotherapy or combination therapy is more effective and safe still needs to be explored. In this study, combination therapy means camrelizumab plus chemotherapy. Chemotherapy drugs include gemcitabine, cisplatin, decitabine, docetaxel, irinotecan, carboplatin, and pemetrexed, which are common and mature chemotherapeutic drugs that are used in strict accordance with the guidelines and instructions. It is of practical significance to study the safety and effectiveness of camrelizumab combined with these chemotherapy drugs and to compare with camrelizumab alone treatment.

Recently, the office of Orphan Products Development (OOPD) of the US Food and Drug Administration (FDA) awarded orphan drug design (ODD) for camrelizumab injection (13). The orphan drug qualification is based on an international multicenter phase III clinical study of first-line treatment of advanced HCC with camrelizumab combined with apatinib in the US, which has been carried out in 13 countries and regions including the US, Europe, South Korea, and China (14). After obtaining the orphan drug qualification, the clinical trials and market registration can be accelerated. Moreover, PubMed and other literature search websites did not find a meta-analysis of the comparison between single drug and combination drug of camrelizumab, so this study has certain importance and innovation. In this study, a total of five types of cancer, HCC, NPC, EC, NHL, and NSCLC, were included. The reason why HCC is not included is that the randomized-controlled trial of camrelizumab combined with apatinib in the treatment of HCC has only published a study protocol, but has not yet produced results (15). This meta-analysis compared the safety and efficacy of camrelizumab monotherapy and combination therapy, aiming to provide a reference for the clinical combined use of camrelizumab in the treatment of cancers and also provide a reference for the development of subsequent indications of camrelizumab.

## Materials and Methods

### Data Sources and Searches

Embase, MEDLINE/Ovid, Epistemonikos, and Cochrane were searched from inception until January 13, 2021. In addition, we also manually searched for articles that met the criteria.

The question for this review was developed using the PICO criteria:

- Population: people aged 18 to 75 years, with NPC, EC, NHL, and NSCLC.
- Intervention: camrelizumab monotherapy or camrelizumab combined chemotherapy.
- Comparator: Camrelizumab + gemcitabine + cisplatin *vs* camrelizumab; camrelizumab + decitabine *vs* camrelizumab; camrelizumab *vs* docetaxel or irinotecan; camrelizumab + carboplatin + pemetrexed *vs* carboplatin + pemetrexed.
- Outcome: ORR, PFS, CR, adverse event rate (grades 1–5).

### Study Selection

We included peer-reviewed systematic reviews with meta-analyses of prospective longitudinal design (i.e., prospective/cohort or retrospective/case-control) studies, as well as randomized-controlled trials (RCTs) that evaluated the efficacy and safety of camrelizumab monotherapy or combined therapy. The criteria are as follows:

Inclusion criteria:

- Meta-analyses that included people aged 18 to 75 years with camrelizumab in a single-drug group or combination therapy group.
- Meta-analyses of prospective longitudinal design studies that explored the efficacy and safety of camrelizumab monotherapy and combined therapy.
- RCTs that investigated the effects and safety of camrelizumab monotherapy and combined therapy.

Exclusion criteria:

- Systematic reviews without meta-analyses.
- Animal or *in vitro* models.
- No peer-reviewed article.
- Conference abstracts.
- Unable to extract valid data.

### Data Screening and Data Extraction

Duplicates exclusion was implemented by two independent reviewers. If there was no consensus, the conflict was solved by a third reviewer. Two independent investigators extracted the following information from each article: (I) publication time; (II) corresponding author and first author; (III) PMID/DOI; (IV) population and main condition of patients in RCT; (V) number of included studies and the total number of people included in the meta-analysis; (VI) study design of included primary studies (e.g., case-control, prospective, RCT); (VII) number of cases and controls for each study; (VIII) mean age of participant population; (IX) primary effectiveness index; (X) primary safety index.

### Risk of Bias and Quality Assessment

The modified Jadad Scoring Scale (16) was used to evaluate the quality of eligible literature methodology with a score system of 1 to 7. Random sequence generation, blind method, randomized allocation concealment, and patient withdrawal were evaluated. Jadad scores of 4 to 7 were high-quality literature, and 1 to 3 were low-quality literature. The Cochrane risk of bias assessment tool was used to assess the methodological quality of individual studies based on the following aspects: random sequence generation, allocation concealment, blinding of participants and personnel, blinding of outcome and assessment, incomplete outcome data, selective reporting, and other bias. Each item was answered with high, low, or unclear risk of bias, and disagreements were resolved through an open discussion or a third reviewer. The general chart of bias risk was made by Revman software.

### Statistical Analysis

Stata 12.0 software was used for meta-analysis. The binary variables were expressed by odds ratio (RR) and 95% confidence interval (CI); the continuous variables were represented by standardized mean difference (SMD) and 95% CI. If there was no statistical heterogeneity among the studies (P > 0.1, I2 < 50%), the fixed-effects model was used for analysis; otherwise, the random effect model was used for analysis. Revman5 software was used to map the risk of publication bias, and Egg’s test was used to analyze publication bias. P < 0.05 was statistically significant.

## Results

### Systematic Literature Search

**Figure 1:**
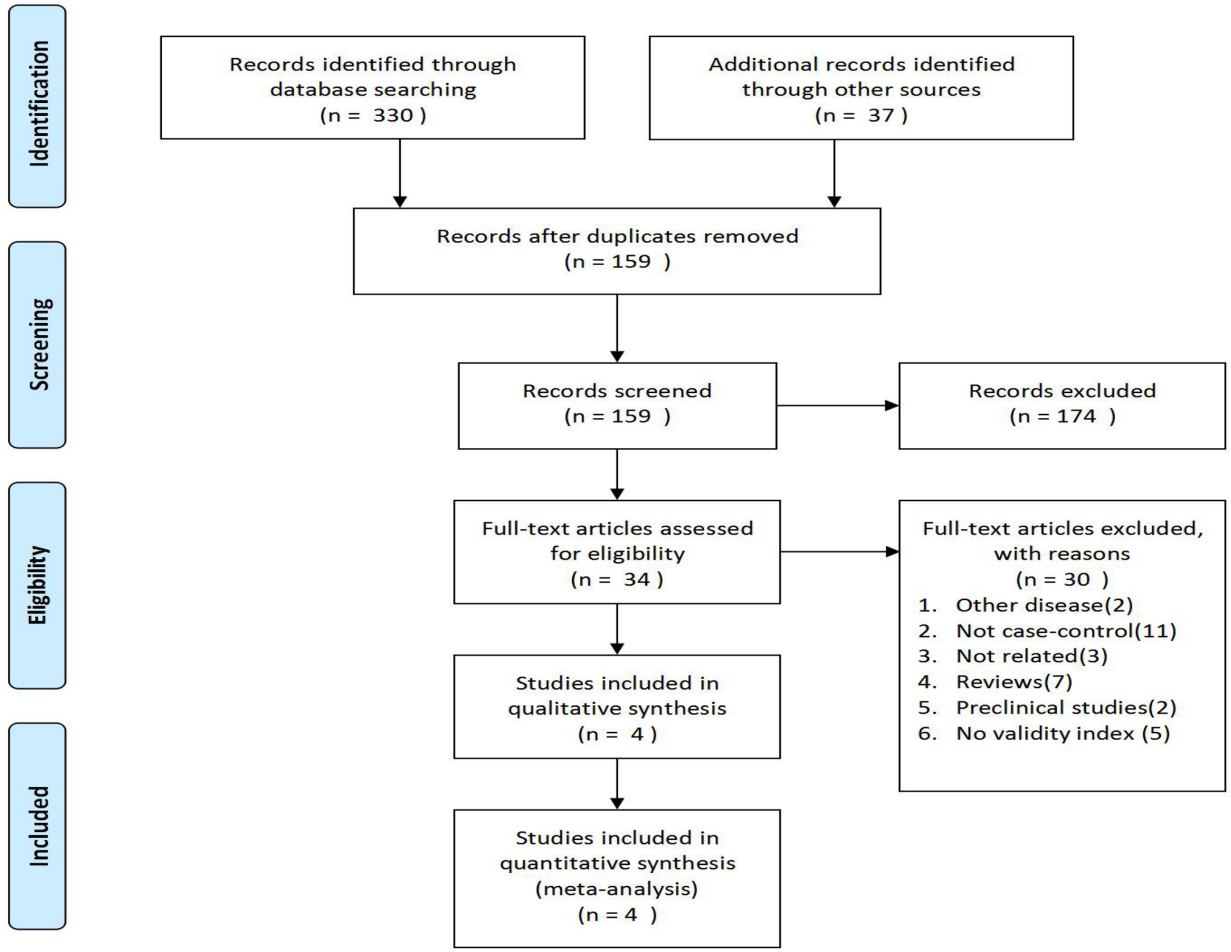
Flowchart of studies evaluating qualified research through selection process.

The flowchart of PRISMA was shown in **Figure 1**. A total of 367 potentially relevant articles were included in the combined electronic and paper reference search. After preliminary screening, 333 publications were excluded according to the title and abstract. After detailed reading and evaluation of the full text, another 30 articles were excluded because they had no valid data to extract, or they were animal or basic research, or they were reviews without meta-analysis, or they were conference abstracts. Overall, four observational studies, involving 1233 patients with camrelizumab monotherapy and combination therapy, were included.

### Study Characteristics and Quality Assessment

The details of the drug interventions, baseline characteristics of the populations, average age, study period, efficacy Index, and safety index of four eligible trials were shown in **Table 1**. The improved Jadad Scale (16) was used to evaluate its quality. The Jadad scores were between five and six points and were all high-quality documents (**Table 1**).

**Table 1:** Characteristics of included studies and Jadad scores.

Study	Jadad	Intervention	n	Year	Study period	Efficacy index and Safety index
Wenfeng Fang (8)	6	Camrelizumab + gemcitabine + cisplatin *vs* Camrelizumab	23/93	18–70	12 months	ORR, PFS, adverse event rate (grade 1-5)
Jing Nie (17)	5	Camrelizumab+ Decitabine *vs* Camrelizumab	42/19	18–75	24 months	ORR, PFS, CR, adverse event rate (grade 1-5)
Jing Huang (18)	6	Camrelizumab *vs* docetaxel or irinotecan	228/220	18–75	24 months	ORR, PFS, CR, adverse event rate (grade 1-5)
Caicun Zhou (19)	6	Camrelizumab + Carboplatin + Pemetrexed *vs* Carboplatin + Pemetrexed	205/207	18–70	22 months	PFS, adverse event rate (grade 1-5)

### Efficacy Analysis

**Figure 2:**
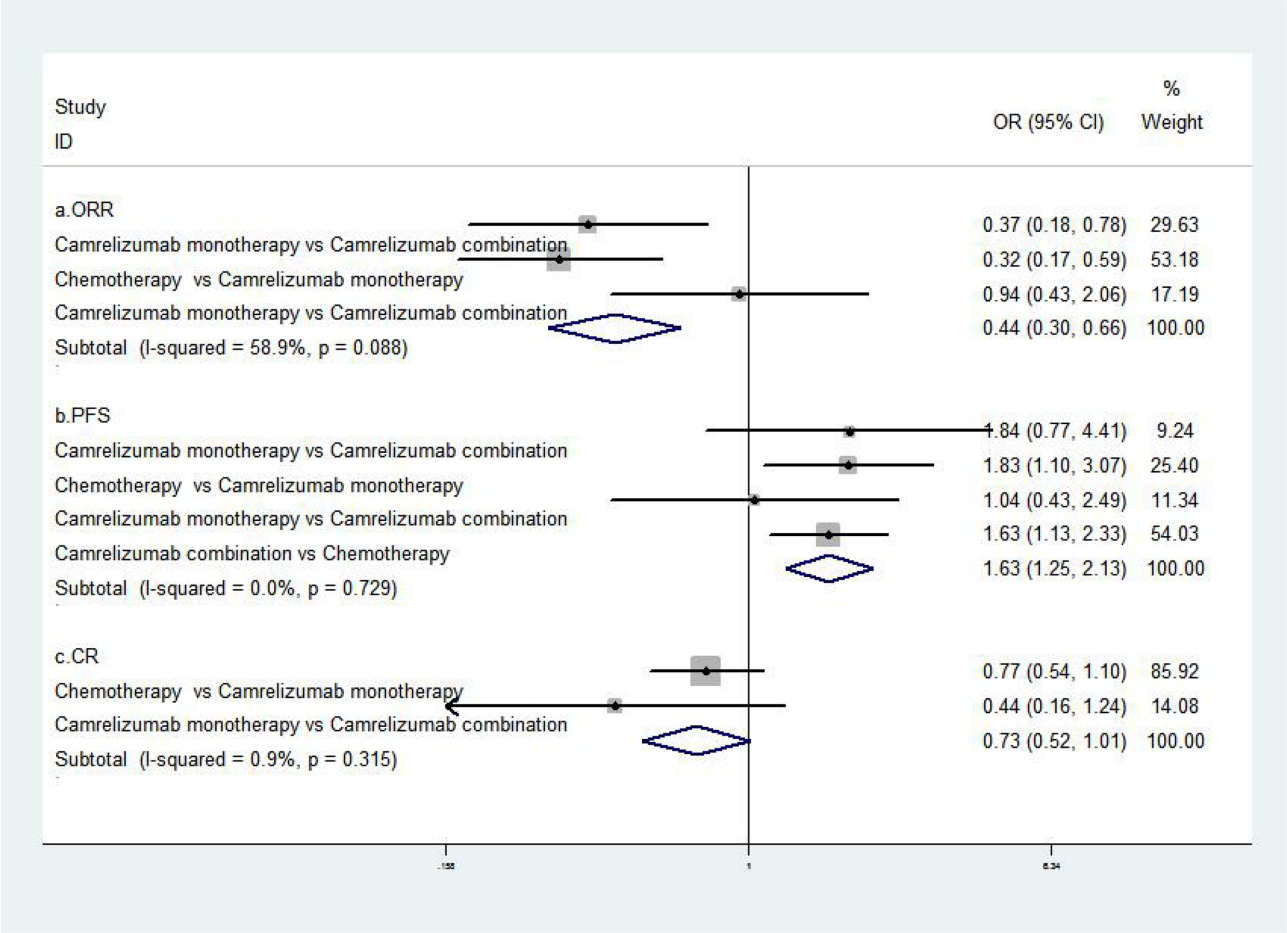
Meta-analysis of efficacy of single and combined treatment of camrelizumab.

The results of the meta-analysis of effectiveness indicators were as follows: the ORR, PFS, and CR value of combined treatment with camrelizumab were better than single camrelizumab treatment, camrelizumab alone was better than chemotherapy [RR=0.44, 95%CI (0.30, 0.66), P<0.001; RR=1.63, 95%CI (1.25, 2.13), P<0.001; RR=0.73, 95%CI (0.52, 1.02), P<0.001] (8, 17–19). The abovementioned results suggested that the combination of camrelizumab was better than monotherapy, and monotherapy was better than chemotherapy (**Figure 2**).

### Safety Analysis

**Figure 3:**
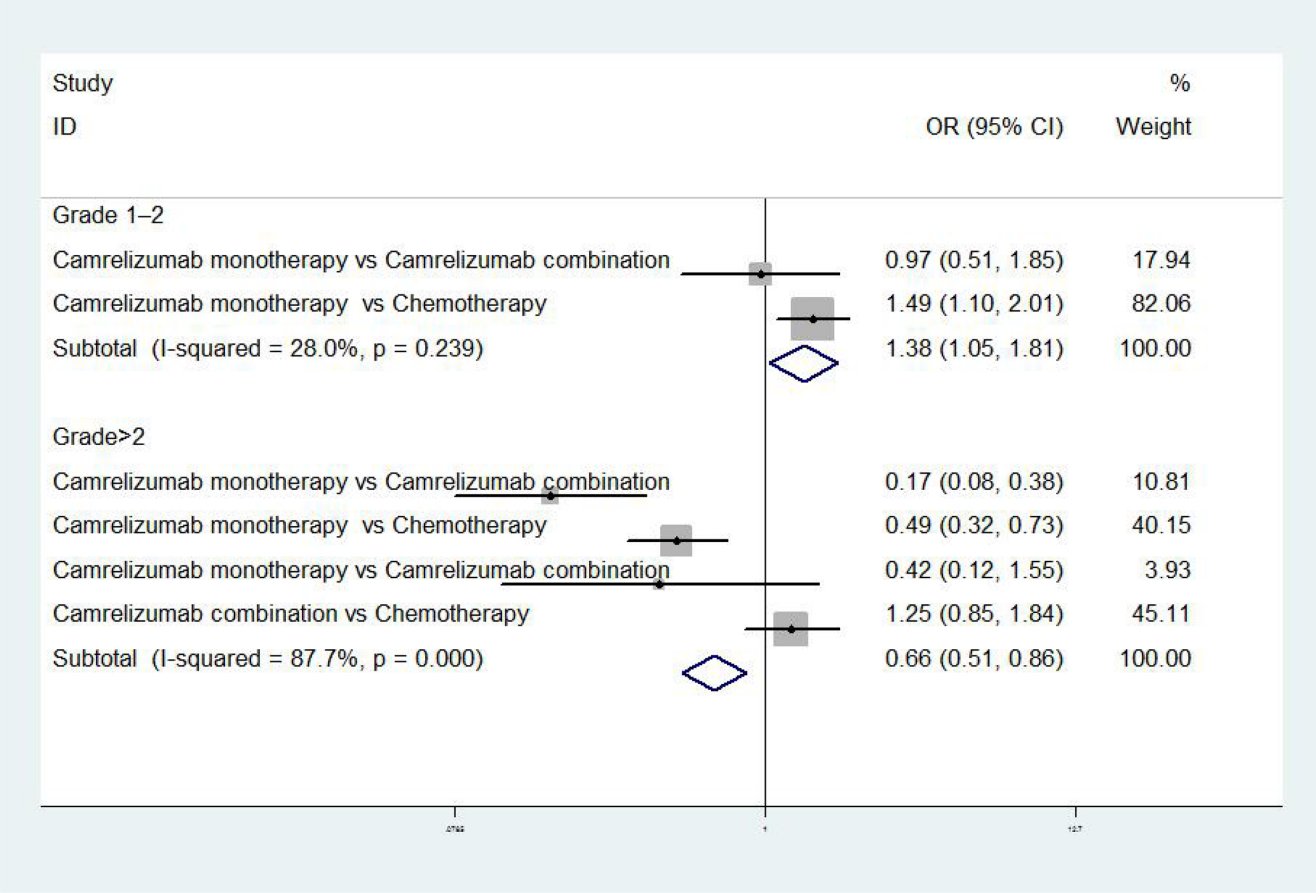
Meta-analysis of safety of single and combined treatment of camrelizumab.

The results of meta-analysis of safety indicators were as follows: when the adverse event was grades 1 to 2, there was no difference in incidence between camrelizumab monotherapy and combination in NPC [OR = 0.97, 95% CI (0.51, 1.85), P>0.01], while the incidence rate of camrelizumab monotherapy was higher than chemotherapy in EC [OR = 1.49, 95% CI (1.10, 2.01), P<0.01] (17, 18). When adverse event was grade>2, the incidence of camrelizumab combination group was higher than chemotherapy [OR = 1.25, 95%CI (0.85, 1.84), P<0.01], meanwhile the incidence of camrelizumab combination group and chemotherapy were higher than camrelizumab monotherapy [OR=0.17, 95%CI (0.08, 0.38), P<0.01; OR=0.49, 95%CI (0.32, 0.73), P<0.01; OR=0.42, 95%CI (0.12, 1.55), P<0.01] (8, 17–19) (**Figure 3**).

Using camrelizumab to treat cancers could cause some common adverse events (any grade), including reactive capillary hyperplasia, elevated aspartate aminotransferase, elevated alanine aminotransferase, anemia, elevated serum bilirubin, leukopenia, and hypothyroidism. From direct comparison, we found that the incidence of reactive capillary hyperplasia, elevated aspartate aminotransferase, elevated alanine aminotransferase, hypothyroidism, elevated serum bilirubin in single drug group was higher than the combination group. The incidence of anemia and leukopenia in the single-drug group were lower than the combination group. The abovementioned results showed that in terms of the incidence of common adverse events (any grade), the safety of combination therapy was better than that of monotherapy (8, 17) (**Table 2**). We also found that the incidence of reactive capillary hyperplasia, elevated aspartate aminotransferase, elevated alanine aminotransferase, anemia, elevated serum bilirubin, leukopenia, hypothyroidism in camrelizumab combination were higher than chemotherapy, suggesting that the safety of chemotherapy was better than camrelizumab combination (19) (**Table 3**).

**Table 2:** Comparison of the incidence of common adverse events (any grade) between camrelizumab alone and combination therapy.

Common adverse event	Camrelizumab combination (incidence rate)	Camrelizumab alone (incidence rate)
	Any grade	Any grade
Reactive capillary hyperplasia	22%	88%
Elevated aspartate aminotransferase	17%	48%
Elevated alanine aminotransferase	14%	48%
Anemia	100%	13%
Elevated serum bilirubin	0%	7%
Leukopenia	87%	12%
Hypothyroidism	4%	16%

**Table 3:** Comparison of the incidence of common adverse events (any grade) between camrelizumab combination and chemotherapy.

Common adverse event	Camrelizumab combination (incidence rate)	Chemotherapy (incidence rate)
	Any grade	Any grade
Reactive capillary hyperplasia	78%	<1%
Elevated aspartate aminotransferase	47%	33%
Elevated alanine aminotransferase	48%	40%
Anemia	34%	27%
Elevated serum bilirubin	11%	5%
Leukopenia	91%	78%
Hypothyroidism	11%	0%

### Heterogeneity and Risk of Bias

**Figure 4:**
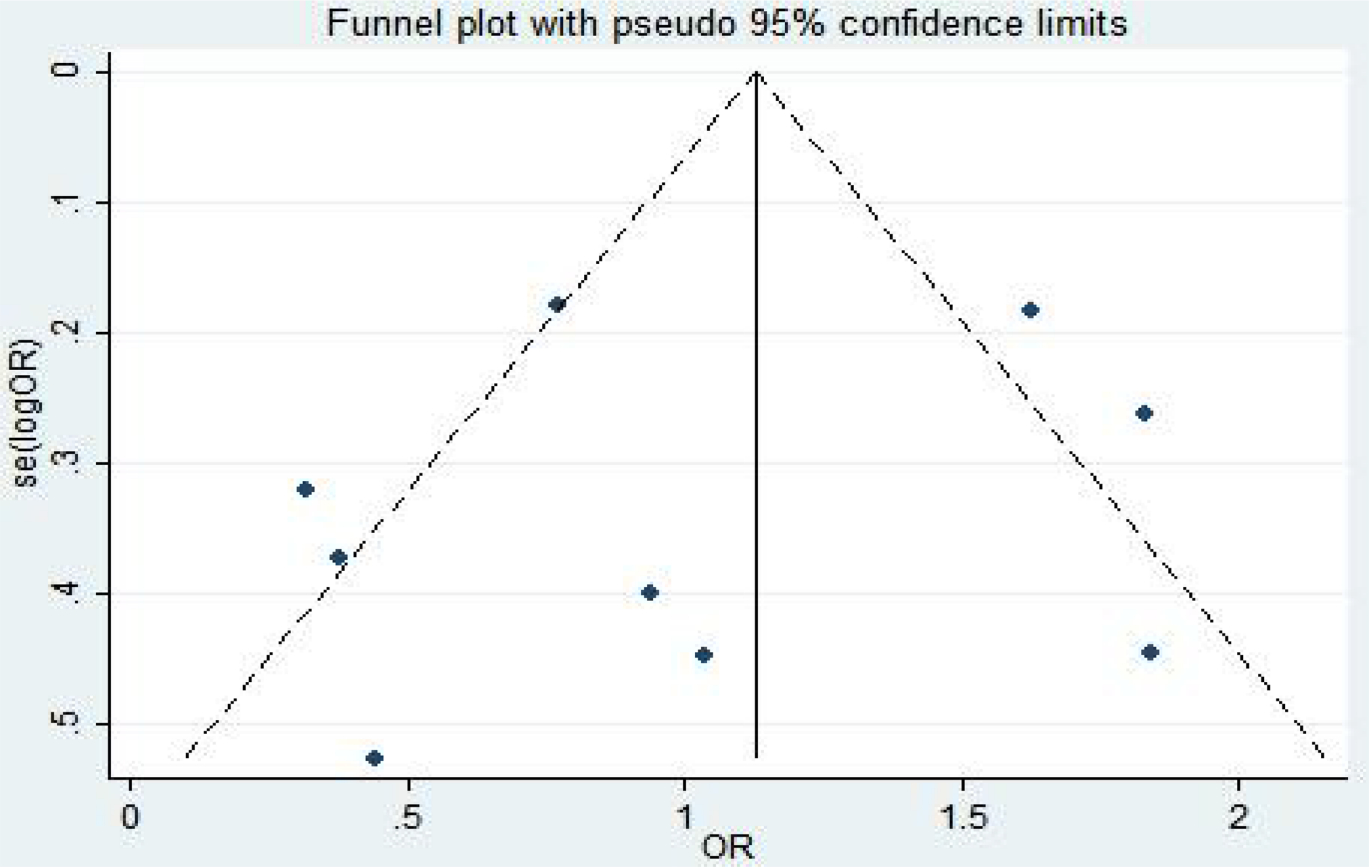
Funnel diagram based on ORR, PFS, CR.

**Figure 5:**
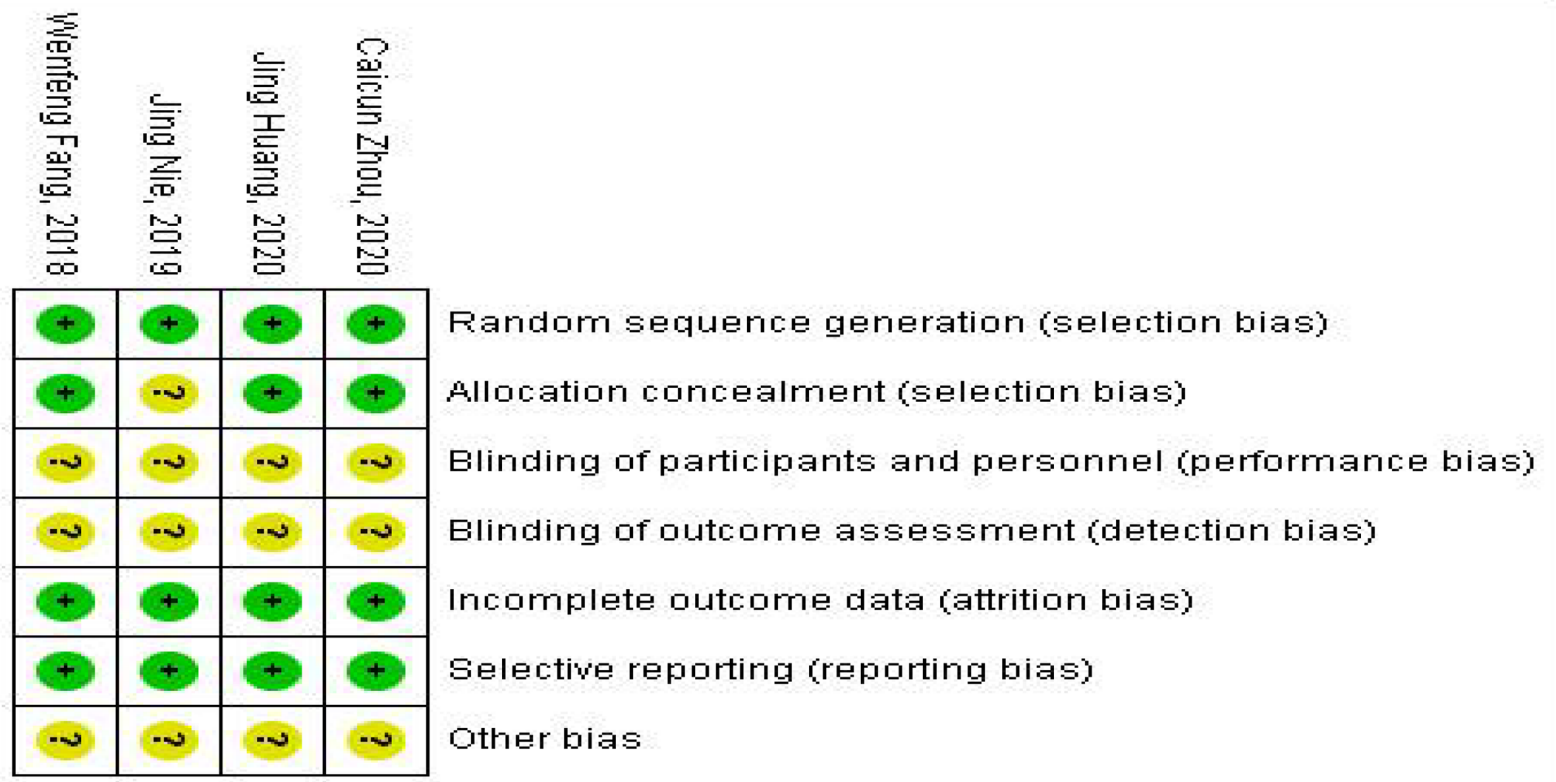
Summary chart of bias risk.

The fixed-effect model was used to analyze the efficacy and safety indicators, and the heterogeneity generally met the requirements. However, the heterogeneity was high at an incidence rate of grade > 2. After the first study was excluded, the heterogeneity could meet the requirements. ORR, PFS, and CR were used as indicators (8, 17–19) for publication bias analysis. Egger’s test was used for publication bias analysis. The standard error of logarithm of OR value of each independent study was taken as abscissa and logarithm of OR value of each independent study as ordinate. A funnel plot was drawn, as shown in **Figure 4**. Egger’s test (P = 0.905 > 0.05) indicated that there was less possibility of publication bias. The general chart of bias risk was shown in **Figure 5**.

## Discussion

Recently, there is now growing worldwide enthusiasm in cancer immunotherapy (20). ICI are the main drugs of immunotherapy and have recently emerged as a promising treatment for many cancers, which identified to target the cytotoxic T lymphocyte-associated antigen-4 (CTLA-4) or PD-1/PD-L1 pathways (21). One of the ICI named camrelizumab (AiRuiKa™), is a PD-1 inhibitor being developed by Jiangsu Hengrui Medicine Co. Ltd (6). In May 2019, camrelizumab for injection was conditionally approved by the State Drug Administration of China for marketing. The indication was the treatment of patients with relapsed or refractory classical HDL who had received at least second-line systemic chemotherapy (12). In 2020, it was approved for the treatment of patients with advanced HCC who had previously received sorafenib treatment and/or oxaliplatin containing systemic chemotherapy, combination of pemetrexed, and carboplatin for the first-line treatment of unresectable locally advanced or metastatic NSCLC with negative epidermal growth factor receptor (EGFR) gene mutation and negative anaplastic lymphoma kinase (ALK), and for the treatment of patients with locally advanced or metastatic ES who had previously received first-line chemotherapy (22, 23). In 2020, camrelizumab was recommended by the Chinese Society of Clinical Oncology (CSCO) clinical guidelines for the treatment of NSCLC, HCC, EC, and HDL and became the only immunotherapy drug to achieve this achievement in China (24). In 2021, camrelizumab was approved by FDA as orphan drug (14). Orphan drug, also known as rare disease drug, refers to the drugs used to prevent, treat, and diagnose rare diseases, the candidate drugs who obtained the orphan drug qualification had the opportunity to obtain a series of supporting policies (25). After obtaining the orphan drug qualification, the clinical trials and market registration can be accelerated, at the same time, the clinical trial fee can be partly offset by tax, the new drug registration fee can be reduced, and the product will enjoy 7 years of market monopoly after being approved (26). Moreover, PubMed, Web of Science, and other literature search websites did not find a meta-analysis of the comparison between single-drug and combination drug of camrelizumab, so this study had certain importance and innovation. Therefore, it can be seen that camrelizumab has a good development prospect and it is necessary to carry out RCTs of single and combined treatment of camrelizumab; however, the RCTs for HCC were in progress, and no clinical results were available, so this meta-analysis included four indications, which were NSCLC, NPC, ES, and HDL.

In the aspect of effectiveness indicators, we found that the ORR, PFS, CR value of combined treatment with camrelizumab were better than that of single-drug treatment, and the ORR, PFS, CR value of single-drug treatment with camrelizumab were better than that of chemotherapy. These results indicated that the combination therapy of camrelizumab was more effective than single drug or chemotherapy (8, 17–19).

In the aspect of safety index, the severity of adverse event was divided into five grades. The most common adverse reactions were reactive capillary hyperplasia (77.4%), elevated aspartate aminotransferase (19.0%), elevated alanine aminotransferase (17.5%), hypothyroidism (16.7%), fatigue (15.3%), anemia (14.1%), elevated serum bilirubin (11.5%), proteinuria (10.8%), fever (10.4%), and leukopenia (10%) (27, 28). Through meta-analysis, we found that when the grade of adverse events was > 2, the incidence of single use was lower than combination therapy and chemotherapy. However, according to the incidence of common adverse reactions (Any grade), the safety of combined use of camrelizumab was better than that of single use, and the safety of chemotherapy was better than the combined use of camrelizumab plus chemotherapy (8, 17–19).

However, this meta-analysis still has some limitations. First, the sample size of RCTs of camrelizumab monotherapy and combination therapy is small because of the lack of existing research. Second, some included studies are lack of double-blind implementation, so there may be selection bias. Finally, although we considered the studies with lower levels of validity, the number was small, and there was the possibility for lack of robustness. Therefore, this conclusion needs to be further confirmed by more high-quality, multi-center, and large-sample researches.

## Conclusion

Overall, in terms of effectiveness, the combination of camrelizumab is better than monotherapy, and monotherapy is better than chemotherapy. In terms of safety, when the grade of adverse events > 2, single use is better than combination therapy and chemotherapy. However, in any grade, the safety of combined use of camrelizumab is better than that of single use, and the safety of chemotherapy is better than the combined use of camrelizumab plus chemotherapy. Therefore, when the patient with serious clinical course and the physical status is tolerable, the combination of camrelizumab is more recommended. When the patient has poor healthy condition, monotherapy is recommended; however, large sample studies are still needed to further prove this inference.

## Data Availability Statement

The datasets presented in this study can be found in online repositories. The names of the repository/repositories and accession number(s) can be found below: Embase, MEDLINE/ Ovid, Epistemonikos and Cochran, accession numbers T5M3L2NLFV,13882707759,20190599120027.

## Author Contributions

JW and YL carried out the conception and design of the article and wrote the paper. JW and JL carried out the implementation and feasibility analysis of the research. JW and SS collected the data. SS carried out the data sorting. JW carried out the statistical processing. JL carried out the analysis and interpretation of the results, and the revision of the paper. YL was responsible for the quality control and review of the article, and analyzed the overall article. All authors contributed to the article and approved the submitted version.

## Funding

This study was supported by the National Natural Science Foundation of China (81803019) and Sichuan Provincial Department of Education (SCYG2019-04, YF19-Y12).

## Conflict of Interest

The authors declare that the research was conducted in the absence of any commercial or financial relationships that could be construed as a potential conflict of interest.
